# Locomotor Ataxy

**Published:** 1883-06

**Authors:** 


LOCOMOTOR ATAXY.

Dr. H. C. Tweedy read a paper on two cases of locomotor ataxy, and exhibited the patients. The first case was that of a pensioner, æt. 64, who was admitted into Stevens' Hospital in 1871, presenting most of the sym,toms of the affection—the peculiar gait, the absence of co-ordination and of the neuralgic pains characteristic of the earlier stages of the disease. He was persistently treated with nitrate of silver, in doses of $\frac{1}{3}$ gr. three times daily, and continued the use of the drug at intervals for nearly twelve years, during which time he was again in the hospital during the years 1673–76–82. The ataxic symptoms had completely disappeared, but from the length of time the silver had been taken the patient had become argyrised. Attention was invited to the peculiar leaden discoloration of the skin from this cause, and the opinion of the members was requested as to whether the symptoms clearly indicated a case of tabes dorsalis, or one of those rare cases in which the progress of the disease had been arrested, and a cure had followed, whether spontaneously or the result of the remedy employed. The second case was that of an engine-driver, æt. 42, in whom the disease was only of six months' standing. This patient also exhibited most of the phenomena of the earlier stage of the disease, the peculiar gait, and fulgurant pains along the course of certain nerves; but in addition there were consecutive attacks of a cutaneous eruption resembling erythema, entirely confined to the left side of the body, and unaccompanied by any of the usually attendant neuralgic pains. There was also a patch of an eruption resembling psoriasis on the back of the left wrist; no similar patch co-existing on the opposite side. Attention was drawn to the fact of eruptions, usually bilateral, appearing only on one side of the body, the connection between these and similar eruptions occurring as trophic lesions in tabes dorsalis, but accompanied invariably by lancinating pain along the course of the nerves over which the eruptions were found.

Dr. Banks, having seen a great many cases of locomotor ataxy, was of opinion that in a considerable number the disease stood still, and in others appeared to be removed. He had used nitrate of silver with great advantage, and did not participate in the terror some had of its effects in producing discoloration of the skin. He had once seen it occur in a case of epilepsy. He believed in the existence of a syphilitic taint in a large proportion of cases.

Dr. Grimshaw remembered the case brought forward by Dr. Tweedy. The result of the treatment was admirable.

Dr. Nixon agreed with Dr. Banks as to the frequency of arrest, and even occasional cure, of the disease, especially in cases in which syphilis existed. He considered the skin affections in one of Dr. Tweedy's cases as coincidences, and preferred referring them to a syphilitic origin.

Dr. Robinson asked whether either of the patients was addicted to the abuse of stimulants.

Mr. Lentaigne mentioned a case in which Langenbeck stretched the sciatic nerve, and the symptoms disappeared. A subsequent autopsy showed the spinal cord to be perfectly healthy.

The President related a case of syphilitic origin which recovered under the use of KI.

Dr. Henry Kennedy and Dr. W. G. Smith also took part in the discussion.

Dr. Tweedy, in replying, said that in the case which had recovered, the man had no syphilitic history. Neither patient had been addicted to intemperance.—*Medical Press.*

---

THE county commissioners have appointed Dr. Wm. T. Belfield Assistant Pathologist at the Cook County Hospital.

Two members of the homœopathic staff have been invited to resign, and their places filled by other similia.

